# Cross-Species Transmission and Recombination Between Feline and Canine Coronaviruses in Jiangsu–Zhejiang Region in 2025

**DOI:** 10.3390/vetsci13070661

**Published:** 2026-07-07

**Authors:** Yanhan Lin, Xiaoyang Zhu, Yifan Meng, Jiachun Zou, Wanying Xie, Shuai Yang, Meng Cui, Ming Qiu, Xinkai Wang, Qinchao Guan, Hong Lin, Sen Jiang, Wanglong Zheng, Jianzhong Zhu, Kewei Fan, Nanhua Chen

**Affiliations:** 1 College of Veterinary Medicine, Yangzhou University, Yangzhou 225009, China; 17757186305@163.com (Y.L.); zhuxiaoyang1998@126.com (X.Z.); m1653385177@163.com (Y.M.); 242002340@stu.yzu.edu.cn (J.Z.); 251706205@stu.yzu.edu.cn (W.X.); ys15238113570@163.com (S.Y.); cuimeng201902@163.com (M.C.); qiuming1997@126.com (M.Q.); 15014175180@163.com (X.W.); 17853406351@163.com (Q.G.); linhonglynn@yzu.edu.cn (H.L.); 008702@yzu.edu.cn (S.J.); wanglongzheng@yzu.edu.cn (W.Z.); jzzhu@yzu.edu.cn (J.Z.); 2 Longyan University and Fujian Provincial Key Laboratory for Prevention and Control of Animal Infectious Diseases and Biotechnology, Longyan 364012, China; 3 Jiangsu Co-Innovation Center for Prevention and Control of Important Animal Infectious Diseases and Zoonoses, Yangzhou University, Yangzhou 225009, China; 4 Comparative Medicine Research Institute, Yangzhou University, Yangzhou 225009, China

**Keywords:** feline coronavirus (FCoV), canine coronavirus (CCoV), universal detection, epidemiology, cross-species transmission, recombination

## Abstract

Feline and canine coronaviruses (FCoVs and CCoVs) are widely prevalent in companion animals. The recent emergence of a highly virulent FCoV-CCoV recombinant virus, FCoV-23, caused a rapid feline infectious peritonitis (FIP) outbreak in Cyprus, which raises a serious concern about the cross-species transmission of CoVs among companion animals. Here, we evaluated the prevalence, transmission and evolution of FCoV and CCoV in the Jiangsu and Zhejiang provinces of China in 2025. A universal RT-qPCR assay for FCoV and CCoV was established and used for the detection of 700 clinical samples, including 501 from cats and 199 from dogs. A total of 92 samples (13.14%, 92/700) were detected as positive, containing 79 positive samples (15.76%, 79/501) from cats and 13 positive samples (6.5%, 13/199) from dogs. Ten complete S genes and two complete genomes were obtained from positive samples. Fragment comparison and phylogenetic analysis identified one CCoV-I sequence from a feline sample and one FCoV-II sequence from a canine sample. Furthermore, recombination analyses not only detected intra-species recombination events but also inter-species cross-over events. Collectively, this study, for the first time, revealed that cross-species transmission and recombination events occurred between FCoV and CCoV among companion animals in the Jiangsu–Zhejiang region of China in 2025.

## Simple Summary

Coronaviruses (CoVs) can infect a wide range of species, including humans, wildlife, livestock and companion animals. Frequent recombination among distinct CoVs facilitates their cross-species transmission. However, the cross-species transmission of CoVs between felines and canines in the Jiangsu–Zhejiang region of China is still rarely estimated. This study developed an RT-qPCR method for the universal detection of feline CoVs (FCoVs) and canine CoVs (CCoVs) in 700 clinical samples collected in 2025. A total of 15.76% (79/501) of cat samples and 6.53% (13/199) of dog samples were detected as positive. Representative positive samples underwent S gene and complete genome sequencing. Multiple alignments and phylogenetic analyses identified CCoV sequences from cat samples and FCoV sequences from dog samples, which supported the existence of cross-species transmission. Intra-species and inter-species cross-over events were detected in our obtained complete genomes, indicating that recombination seems to contribute to the cross-species transmission of CoVs between cats and dogs. Overall, this study provides the first evidence supporting the occurrence of cross-species transmission and recombination events in companion animals in the Jiangsu–Zhejiang region of China in 2025.

## 1. Introduction

Coronaviruses (CoVs) are positive-sense single-stranded RNA viruses with some of the largest genome sizes of ~30 kb and belong to the *Coronaviridae* family within the *Nidovirales* order [1]. The genome of CoVs contains two large open reading frames (ORFs) encoding two polyproteins (pp1a and pp1ab) critical for viral replication. Nine smaller ORFs following ORF1b encode four structural proteins (spike (S), envelope (E), membrane (M), and nucleocapsid (N)) and five accessory proteins (3a, 3b, 3c, 7a, 7b) [2,3]. CoVs exhibit broad host tropism, which can not only infect humans but can also infect almost all animal species, including wildlife, livestock and companion animals (especially cats and dogs) [4,5]. Feline coronavirus (FCoV) can infect wild and domestic cats, with ~12% of infections resulting in deadly feline infectious peritonitis (FIP) [6]. Canine coronavirus (CCoV) is a common enteric pathogen in dogs, mainly affecting neonatal and juvenile puppies, causing vomiting, diarrhea and dehydration [7]. Two recent studies published in *Nature* reported the emergence of a highly pathogenic FCoV-CCoV recombinant (FCoV-23) responsible for an outbreak of FIP in Cyprus [4,8]. Within the One Health paradigm, the health of companion animals is closely linked to human health [7,9]. Notably, FCoV and CCoV are both clustered within the *Alphacoronavirus* genus, which also contains CoVs that can infect humans (HCoV-229E) [10]. The widespread prevalence of FCoV and CCoV not only affects the health of companion animals but may also become a potential source of cross-species transmission, resulting in virus spillover [1,5,9]. Therefore, up-to-date studies on the cross-species transmission and recombination potential of FCoV and CCoV are of paramount importance [7,11].

FCoV can be distributed into two phylogenic lineages: FCoV-I and FCoV-II [12]. FCoV-1 has widely circulated in the world, accounting for 80~95% of natural infections, while FCoV-II is sporadically detected, originating from the recombination of FCoV-I and CCoV [13]. Intriguingly, FCoV-I has demonstrated a fastidious nature in cell culture growth, but FCoV-II exhibits adaptability to cell culture systems [14]. Due to mutations in the S gene and accessory genes, FCoV can change its cell tropism from the enteric tract to macrophages, leading to distinct disease presentations in two biotypes: feline enteric coronavirus (FECV) and feline infectious peritonitis virus (FIPV) [4]. FECV is a predominant and avirulent biotype that commonly causes mild enteric or respiratory symptoms [11]. FIPV is the highly virulent biotype and is responsible for severe pathological outcomes, inducing effusive peritonitis, neurological dysfunction, and ocular lesions [15]. Based on the divergence of the S gene, CCoV can be clustered into two genotypes: CCoV-I and CCoV-II [7]. Some new CCoV-II variants are associated with systemic and fatal disease owing to genetic drift or recombination, which can be further divided into two sub-genotypes (CCoV-IIa and CCoV-IIb) [16]. Currently, intra-species and inter-species transmission and recombination of distinct FCoV and CCoV strains have been globally reported [1,4,16,17].

Occurrences of FCoV and CCoV infections are frequently observed in China; FCoV may cause feline infectious peritonitis (FIP), a severe systemic disease with an extremely high mortality rate in the absence of effective treatment. Furthermore, CCoV-FCoV recombinant strains may facilitate rapid FIP outbreaks, leading to high mortality in affected feline populations [4,18]. However, studies on the epidemiology and transmission of FCoV and CCoV in China are still rare. Here, for the first time, we developed a universal RT-qPCR assay for the detection of both FCoV and CCoV. Then, the prevalence of FCoV and CCoV in the Zhejiang and Jiangsu provinces of China in 2025 was evaluated using 700 clinical samples, including 501 feline samples and 199 canine samples. Ten complete S genes and two complete genomes (one FIPV and one FECV) were determined and used for genetic evolution and recombination estimation. The results of this study provide up-to-date information on the epidemiology and evolution of FCoV and CCoV in the Jiangsu–Zhejiang region of China.

## 2. Materials and Methods

### 2.1. Establishment of a Universal RT-qPCR Detection Assay for Feline–Canine CoVs

A total of 100 FCoV (60 representative sequences) and CCoV (40 representative sequences) complete genomes were retrieved from the National Center for Biotechnology Information (NCBI) nucleotide database, and sequence alignment was performed using the SnapGene software, version 7.1.2. A highly conserved 3′UTR region of FCoVs and CCoVs was selected for primer and probe design. A TaqMan-MGB probe with a FAM fluorophore at the 5′ end and pairing primers were designed for both FCoV and CCoV strains (Table 1). In addition, to prepare a standard positive control, a plasmid containing the 3′UTR sequence was constructed using the pMDTM18-T Vector Cloning Kit (TaKaRa, Osaka, Japan) and confirmed by Sanger Sequencing at the GENEWIZ Company (Suzhou, China). The recombinant plasmid was purified with the FastPure Plasmid Mini Kit (Vazyme, Nanjing, China) and quantified using the Nano200 spectrophotometer (Aosheng, Hangzhou, China). The concentration was converted into copy number using the formula y (copies/μL) = (6.02 × 10^23^) × (x (ng/μL) × 10^−9^ DNA)/(DNA length × 660), as we previously described [19]. The plasmid was diluted to obtain a stock solution of 10^9^ copies/μL. The standard curve was generated by using 10-fold serial dilutions of the standard plasmid.

To estimate the specificity of the universal RT-qPCR assay, cDNA from FCoV and CCoV and cDNA/DNA from other pathogens, including feline immunodeficiency virus (FIV), feline calicivirus (FCV), feline herpesvirus 1 (FHV-1), feline panleukopenia virus (FPV), and Tritrichomonas foetus, were evaluated. The sensitivity of the universal RT-qPCR assay was determined by testing a series of 10-fold diluted standard plasmids. Repeatability (intra-assay) and reproducibility (inter-assay) of the universal RT-qPCR assay were evaluated using three different concentrations (10^7^, 10^5^, 10^3^ copies/μL), as we previously described [19]. To further validate the universal RT-qPCR assay, the same FCoV-positive samples identified by our universal RT-qPCR assay were also submitted to detection under another FCoV RT-qPCR assay as described previously [20].

### 2.2. Clinical Sample Collection

A total of 700 clinical samples were collected from Zhejiang and Jiangsu provinces of China in 2025, including 501 feline samples and 199 canine samples. There were 655 samples from three large companion animal referral and treatment hospitals in Zhejiang province, and 45 samples were collected from 7 animal hospitals in Jiangsu province. The sample types included sera (463), feces (152), nasal–oral swabs (58), ascitic fluids (17) and pleural fluids (10). The 700 samples were obtained from 501 cats and 199 dogs brought to the hospitals for disease treatment, routine health examination, or boarding services. All the clinical samples were collected by the veterinarians at the animal hospitals and sent to our laboratory for detection on ice packs or dry ice.

### 2.3. RNA Extraction and RT-qPCR Detection

Total RNA was extracted from clinical samples, including sera, feces, oral–nasal swabs, ascitic fluids and pleural fluids that were collected from cats and dogs using TRIpure Reagent (Aidlab, Beijing, China). Corresponding cDNA was generated by reverse-transcription using the HiScript III 1st Strand cDNA Synthesis Kit (+gDNA wiper, Vazyme, Nanjing, China). The obtained sample cDNA was detected by our newly developed universal RT-qPCR detection assay using qPCR Premix Taq^TM^ (TaKaRa, Osaka, Japan) and the StepOne Plus Real-Time PCR System (Thermo Fisher Scientific, Waltham, MA, USA) [19]. The 20 μL reaction mixture contained 10 μL Premix Taq, 0.5 μL each primer (10 μM), 0.4 μL probe, and 2 μL viral cDNA or standard DNA. The amplification condition was set at 95 °C for 30 s, followed by 40 cycles of 95 °C for 5 s and 60 °C for 1 min [19,21]. The RT-qPCR amplicons were verified by Sanger Sequencing at Jiangsu Kangwei Century Biotechnology Co., Ltd. (Suzhou, China).

### 2.4. Genome Sequencing and Sequence Comparison

Positive clinical samples underwent complete S gene sequencing using primers shown in Table 1. In addition, two representative FIPV- and FECV-positive samples underwent complete genome sequencing, as previously described [22,23]. Our complete FIPV and FECV genomes were compared with representative CoV genomes (BLACK (EU186072), C1Je (DQ848678), HF1902 (MT444152), UG-FH8 (KX722529), SD (MW030110), JP15 (LC742526), FIPV79-1146 (DQ010921)) using DNAMAN [24,25]. The ten S genes and two complete genomes obtained in this study have been submitted to the GenBank database under accession numbers PZ518627~PZ518638. The sequence nomenclature follows the following format: virus type—sample collection year—sampling location—animal type—sample ID—sample type. For instance, the FECV-25HZ-F6-Fec indicates an FECV collected in 2025 from Hangzhou in feline sample no. 6 in feces. Samples designated as FIPV were all obtained from cats with FIP clinical signs. These cats completed the clinical diagnostic procedure for FIP confirmation, and the pleural and ascitic effusions were collected during the active disease stage. Samples labeled as FECV were derived from cats with no clinical manifestations of FIP; routine samples (serum, feces, and nasal–oral swabs) rather than pleural or ascitic effusions were collected. Samples annotated as CCoV were of canine origin.

### 2.5. Phylogenetic Analyses and Recombination Detection

To estimate the evolutionary relationships of our FCoV and CCoV sequences with other CoVs, a total of 50 CoV complete genomes (48 representative genomes and our 2 genomes) and 58 S genes (48 representative S genes and our 10 S genes) were used for multiple sequence alignment by ClustalW. Genome-based and S-based phylogenetic trees were constructed via MEGA12 using the maximum-likelihood method. The robustness was evaluated by bootstrapping using 1000 replicates, as described previously [26]. To evaluate the potential cross-over events in our obtained complete genomes, multiple aligned genomes were also submitted to recombination screening using recombination detection program 4.0 (RDP4.0) and SimPlot 3.5.1, as we previously described [21,24,25].

## 3. Results

### 3.1. Development of a Universal RT-qPCR Assay for FCoV and CCoV

**Figure 1:**
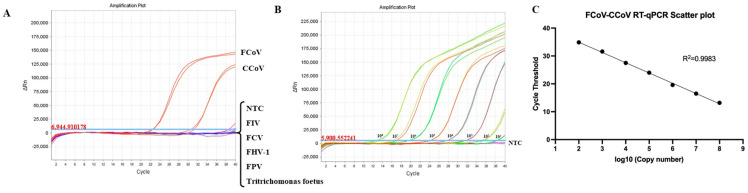
Specificity and sensitivity evaluations of the universal FCoV-CCoV RT-qPCR assay. (**A**) FAM fluorescent signals were observed only when FCoV and CCoV were detected but not for the other pathogens. (**B**) The detection limit of this universal RT-qPCR assay was 10^2^ copies/μL. (**C**) Standard curve for FCoV qPCR. Cycle threshold (Ct) values are plotted against log_10_-transformed viral copy numbers of serially diluted standards, with a coefficient of determination (R^2^) of 0.9983.

A highly conserved region in the 3′UTR of the FCoV and CCoV genomes was chosen to design the CoV-F2/CoV-R2 primer pair and CoV-P0 probe (Table 1). To validate the specificity of the newly developed RT-qPCR assay for FCoV and CCoV, besides representative FCoV- and CCoV-positive samples, clinical samples positive for FIV, FCV, FHV-1, FPV, and Tritrichomonas foetus (all these samples were from clinically confirmed diseased cats that tested negative for FCoV and CCoV) were tested, which showed that FAM fluorescent signals could only be detected in FCoV- and CCoV-positive samples but not in all the other samples (Figure 1A). The sensitivity was tested using 10-fold serial dilutions of a standard plasmid, which showed that the limit of detection (LOD) was 10^2^ copies/μL (Figure 1B). The standard curve showed a strong linear correlation with an *R*^2^ of 0.9983, validating the reliability of this assay (Figure 1C). The repeatability and reproducibility were also evaluated, which showed that the coefficients of variation (CV) for both intra-assay repeatability and inter-assay reproducibility were less than 2% (Table 2). In addition, a cycle threshold (Ct) value < 37 was set as the positive cutoff in sample detection. All these results supported the satisfactory specificity, sensitivity, repeatability and reproducibility of this newly developed universal RT-qPCR assay for FCoV and CCoV.

### 3.2. Clinical Sample Detection

A total of 700 clinical samples were tested by this newly developed RT-qPCR assay (Table 3). The overall positive rate was 13.14% (92/700) for all clinical samples, within which the FCoV-positive rate was 15.76% (79/501), while the CCoV-positive rate was 6.5% (13/199). In detail, the positive rates of samples collected from Zhejiang and Jiangsu were 11.45% (75/655) and 37.77% (17/45), respectively. Meanwhile, the positive rates of different sample types, including sera, feces, nasal–oral swabs, ascitic fluids and pleural fluids, were 6.91% (32/463), 24.34% (37/152), 3.34% (2/58), 88.23% (15/17) and 60% (6/10), respectively. To further validate the practicability of our newly developed RT-qPCR assay, the FCoV-positive samples identified by our assay were also detected by a previous FCoV detection method [20]. Only 58 samples (11.57%, 58/501) were detected as FCoV positive in the previous method, while 79 samples (15.76%, 79/501) were detected as FCoV positive in our assay (Table S1). All these positive samples were confirmed by Sanger sequencing, supporting the satisfactory detection ability of our assay. All of these results indicated that FCoVs and CCoVs co-existed in the feline and canine populations in the Zhejiang and Jiangsu provinces of China in 2025.

### 3.3. Complete Genome and S Gene Sequencing

**Figure 2:**
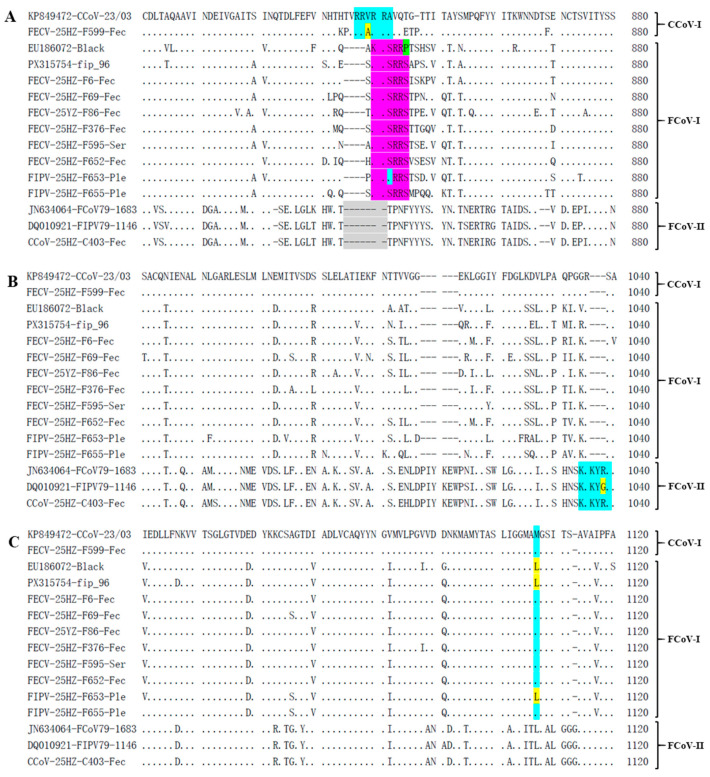
Multiple alignments of S fragments. (**A**) The FECV-25HZ-F599-Fec strain harbored the RRARRA furin cleavage motif. The remaining eight FCoV strains possessed canonical FCoV-I RRS/ARRS furin cleavage sequences at the S1/S2 boundary. The CCoV-I isolate CCoV-25HZ-C403-Fec lacked a canonical S1/S2 furin cleavage site, which shared identical cleavage patterns with FCoV-II reference sequences. (**B**) The CCoV-25HZ-C403-Fec sequence lacked a typical S1/S2 furin cleavage site. Instead, it had a KRKYRS cleavage sequence at the S2′ position. (**C**) Among FCoV-I strains, the FIPV-25HZ-F653-Ple strain harbored the M1058L mutation, while the FIPV-25HZ-F655-Ple retained the original methionine (M) residue. The key residues are highlighted in distinct colors.

Full-length S genes were successfully amplified from 10 positive clinical samples, including nine FCoV strains and one CCoV strain. The lengths of S genes from the nine FCoV strains ranged from 4404 bp to 4449 bp, while the single CCoV S gene was 4362 bp (PZ518627~PZ518636). In Table 2, multiple alignment of our 10 S proteins and five reference S proteins revealed distinct S1/S2 furin cleavage site motifs: one FCoV strain (FECV-25HZ-F599-Fec) harbored the RRARRA furin cleavage motif, which is consistent with the signature S1/S2 site characteristic of CCoV-I serotype [27]. The remaining eight FCoV strains (FECV-25HZ-F6-Fec, FECV-25HZ-F69-Fec, FECV-25HZ-F86-Fec, FECV-25HZ-F376-Fec, FECV-25HZ-F595-Ser, FECV-25HZ-F652-Fec, FIPV-25HZ-F653-Ple, and FIPV-25HZ-F655-Ple) possessed canonical FCoV-I RRS/ARRS furin cleavage sequences at the S1/S2 boundary [28]. Meanwhile, the CCoV isolate CCoV-25HZ-C403-Fec lacked a canonical S1/S2 furin cleavage site, matching the genetic feature of FCoV-II [27] (Figure 2A). The CCoV-25HZ-C403-Fec lacked a typical S1/S2 furin cleavage site. Instead, it contained a KRKYRS cleavage sequence at the S2′ position (Figure 2B), which matched the conserved signature motif of the FCoV-II strains. Among the FCoV-I strains, the FIPV-25HZ-F653-Ple strain harbored the M1058L mutation, consistent with the BLACK and FIP_96 strains [29]. By contrast, the FIPV-25HZ-F655-Ple strain retained a methionine (M) residue at this position (Figure 2C).

To further characterize the genomic profiles of these strains, one FIPV (FIPV-25HZ-F653-Ple) and one FECV (FECV-25HZ-F652-Fec) were submitted to complete genome sequencing; the obtained whole genomes have been deposited in GenBank under accession numbers PZ518637 and PZ518638, respectively. As summarized in Table 4, the FIPV-25HZ-F653-Ple and FecV-25HZ-F652-Fec genomes shared 91.59% homology. However, they shared only 83.78% and 82.82% genome identity with the prototype FCoV-II virulent strain FIPV79-1146, respectively. Notably, FIPV-25HZ-F653-Ple and FECV-25HZ-F652-Fec exhibited high genome similarities (91.94% and 91.13%) with the FCoV-I Denmark strain UG-FH8. Segment-by-segment pairwise comparisons indicated that the spike (S) protein represented the most divergent structural protein, whereas 3c was the most variable non-structural protein (Table 4).

### 3.4. Phylogenetic Analysis

**Figure 3:**
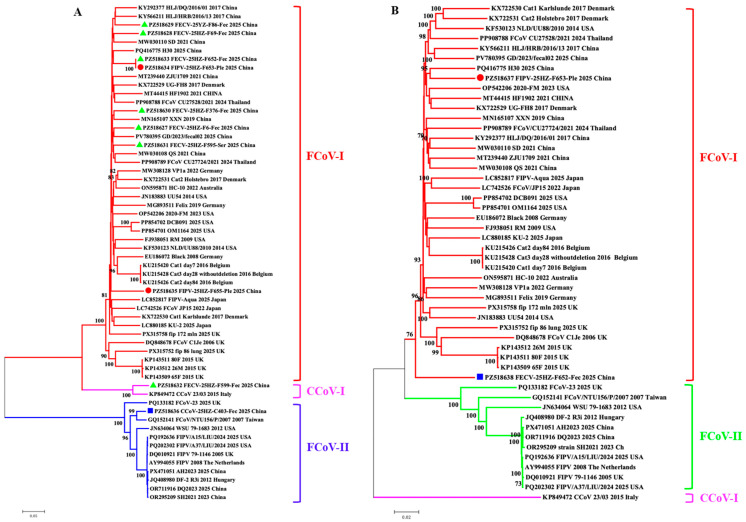
Phylogenetic analyses based on S and genome sequences. (**A**) S-based phylogenetic tree. FECV-25HZ-F599-Fec was clustered within the CCoV-I group, CCoV-25HZ-C403-Fec belonged to FCoV-II, and the other eight S sequences were clustered with FCoV-I. (**B**) Genome-based phylogenetic tree. Both FIPV-25HZ-F653-Ple and FECV-25HZ-F652-Fec belonged to the FCoV-I subgroup. Each subgroup is shown in a distinct color. The obtained S genes and complete genomes in this study are highlighted.

To evaluate the evolutionary relationships among the 10 obtained S genes and reference FCoV/CCoV strains, phylogenetic trees based on complete S sequences were constructed (Figure 3). The tree topology divided all sequences used in this study into three distinct clades: FCoV-I, FCoV-II, and CCoV-I. Eight of our S sequences were grouped with the FCoV-I clade, while FECV-25HZ-F599-Fec was clustered in the CCoV-I clade, and CCoV-25HZ-C403-Fec was grouped with the FCoV-II clade (Figure 3A).

To further analyze the phylogenetic relationships between our genome sequences (FIPV-25HZ-F653-Ple and FECV-25HZ-F652-Fec) and global FCoV/CCoV reference strains, a genome-based phylogenetic tree was also constructed, which revealed that both FIPV-25HZ-F653-Ple and FECV-25HZ-F652-Fec were clustered within the FCoV-I clade. Notably, FIPV-25HZ-F653-Ple shared close genetic relatedness with previously reported Chinese FCoV isolates. However, FECV-25HZ-F652-Fec formed an independent, distinct subclade, suggesting the potential existence of recombination (Figure 3B).

### 3.5. Recombination Evaluation

RDP4 was used to detect cross-over events in our strains. The FECV-25HZ-F652-Fec strain was generated via intra-species recombination, with its genome derived from three recombination events. The first cross-over event occurred in the ORF1a region (5970–8788 bp), which were derived from the FCoV-I NLD/UU88/2010 strain; the second recombination event was located in ORF1b (13,914–15,042 bp), whose fragment was from the FCoV-I HF1902 strain; the last cross-over event spanned ORF3c, E, M, and N (25,412–27,350 bp), and it was inherited from the FCoV-I FIPV-25HZ-F653-Ple strain (Table 5).

**Figure 4:**
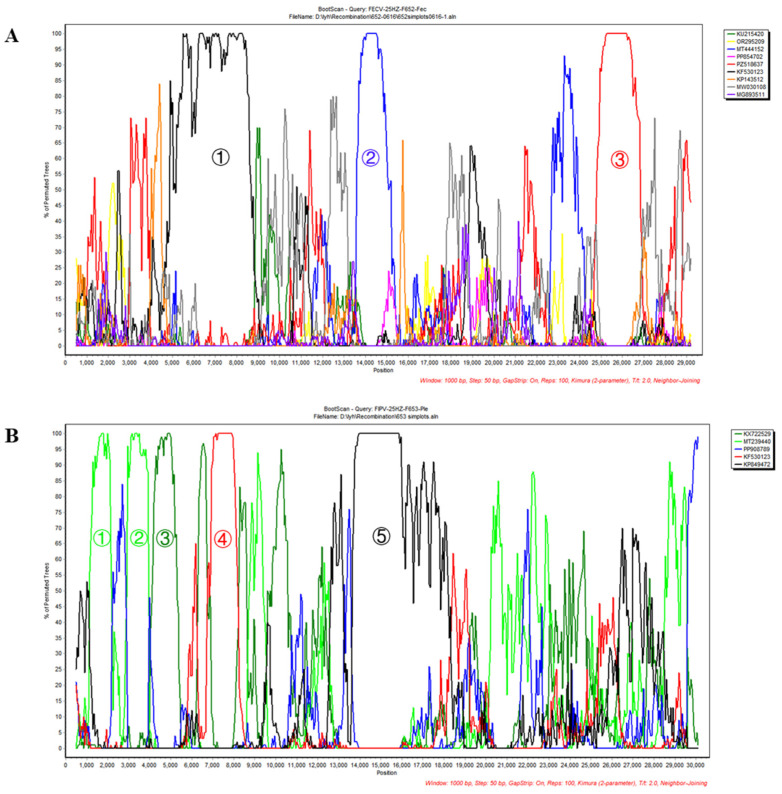
Recombination events in FECV-25HZ-F652-Fec and FIPV-25HZ-F653-Ple. The cross-over regions were detected by SimPlot 3.5.1 and were basically consistent with the results from the RDP4 analysis. (**A**) Three cross-over events in the generation of the FECV-25HZ-F652-Fec strain. (**B**) Five recombination events in the generation of the FIPV-25HZ-F653-Ple strain. The y-axis shows the percentage of permutated trees employing a sliding window of 1000 bp and a step size of 50 bp.

In contrast, the FIPV-25HZ-F653-Ple strain stemmed from inter-species recombination between feline and canine coronaviruses. Its ORF1b fragment (14,006–15,004 bp) was derived from the CCoV-I 23/03 strain, whereas the ORF1a region consisted of four recombinant segments originating from three FCoV-I strains: the ZJU1709 strain contributed sequences at 1543–2230 bp and 2813–3696 bp, while the UG-FH8 strain and NLD/UU88/2010 strain provided fragments at 4504–5090 bp and 7028–8220 bp, respectively (Table 5). The cross-over events in both the FECV-25HZ-F652-Fec and FIPV-25HZ-F653-Ple strains were further confirmed by SimPlot analyses (Figure 4).

## 4. Discussion

Recent studies have confirmed that canine–feline–porcine-like (CFPL) CoVs of the *Alphacoronavirus* 1 species can infect humans and are associated with acute respiratory illness [9,30], attracting worldwide attention to the cross-species transmission and recombination of FCoV and CCoV. However, studies on the prevalence, transmission and evolution of FCoV and CCoV in China have been limited in recent years. In this study, we developed a universal RT-qPCR assay for the rapid detection of FCoV and CCoV in companion animals. Using this new assay, we investigated the prevalence of FCoV and CCoV in the Zhejiang and Jiangsu provinces of China in 2025. In addition, representative S genes and full-length genomes were determined to estimate the transmission and evolution of FCoV and CCoV in the Zhejiang–Jiangsu region in 2025. Our results support the existence of cross-species transmission of FCoV and CCoV in China in recent years.

New CoVs keep emerging all the time; therefore, the corresponding diagnostic methods need to be updated according to distinct purposes. Several PCR detection methods have been developed, mainly focusing on a single CoV-targeting 3′UTR, S gene or nsp14 gene [20,31,32]. For universal detection of FCoV and CCoV, this study developed a 3′UTR-targeted RT-qPCR assay. Comparing the detection of FCoV-positive clinical samples confirmed that this newly developed assay exhibited superior practicability compared with a previously developed method [20]. Therefore, here, we provide an alternative assay for rapid and universal detection of FCoV and CCoV in companion animals.

Both FCoV and CCoV are widely prevalent all around the world. In China, FCoV is highly prevalent in northern China (80.0%), southern China (73.1%), southwestern China (80.35%), eastern China (74.6%), and central China (46.6%) [19,33]. A recent study from the Guangxi province of China showed that the FCoV-positive rate was 17.66% (330/1869) during 2021–2024 [13]. The prevalent rate of CCoV in five provinces of China (Guangdong, Zhejiang, Heilongjiang, Jiangsu and Anhui) was 23.94% (51/213) [34]. Another two studies in Chengdu City, China, from 2020 to 2021 showed that CCoV-positive rates were 27.1% (59/218) and 34.2% (40/117) [32,35]. In this study, we evaluated the prevalence of FCoV and CCoV in the Zhejiang–Jiangsu region of China in 2025. Our results showed that FCoV- and CCoV-positive rates were 15.76% and 6.50%, respectively. Notably, ascitic and pleural fluids had obviously higher positive rates (88.23% and 60%) than serum samples and nasal–oral swabs (6.91% and 3.44%). Therefore, the relatively low positive rates in this study compared with previous studies were likely associated with the high percentages of serum samples (66.14%, 463/700) and nasal–oral swabs (8.28%, 58/700) used in this study, both of which had low positive rates (6.91% and 3.44%).

Mutations in S protein cleavage sites are critical determinants of coronavirus tropism and pathogenesis [27,28]. Proteolytic cleavage is critical for activating the fusion function of the S protein by triggering fusion peptide release, following the initial receptor-binding step. Coronavirus S proteins typically contain multiple proteolytic cleavage sites: the first identified site lies at the S1/S2 subunit boundary, and a second functionally important site within the S2 subunit is termed the S2′ site. Through S gene alignment, we confirmed that eight strains showed the characteristic cleavage pattern of FCoV-I, supporting that FCoV-I strains are the predominant circulating viruses in China [18,28,29,36]. One FCoV strain exhibited identical S protein sequences and S1/S2 cleavage profiles with CCoV-I [27]. One CCoV strain lacked the S1/S2 cleavage site but harbored the conserved S2′ cleavage motif, matching the hallmark signature of FCoV-II [28]. The M1058L mutation is primarily associated with viral tissue tropism and pathological changes [13,37]. Two of the eight FCoV-I strains were FIPV strains, among which only FIPV-25HZ-F653-Ple carried the M1058L amino acid substitution [37]. Collectively, these findings indicated that the FCoV S genes from the Zhejiang–Jiangsu region exhibit a high degree of genetic diversity.

Phylogenetic analysis showed that all FCoV sequences (nine S genes and two complete genomes) obtained in this study belonged to FCoV-I, which is consistent with a previous study showing that all 63 FCoV strains obtained in their study clustered within FCoV-I [13]. Both CCoV-I and CCoV-II circulate in China [32,35,38]. Only one CCoV S sequence (CCoV-25HZ-C403-Fec) was obtained in this study. However, S-based phylogenetic analysis showed that CCoV-25HZ-C403-Fec clustered within the FCoV-II clade. In contrast, an FCoV S sequence (FECV-25HZ-F599-Fec) clustered with the CCoV-I strain (Figure 3A). The phylogenetic results were also consistent with the S protein comparison (Figure 2A), suggesting the occurrence of cross-species transmission and recombination of FCoV and CCoV.

Recombination plays a critical role in the generation of novel CoVs. A highly pathogenic FCoV-CCoV recombinant was responsible for a rapid outbreak of FIP in Cyprus [4]. A novel canine-like *aphacoronavirus* (CCoV-HuPn-2018) probably recombined from canine, feline and porcine *alphacoronaviruses* and was closely associated with acute respiratory illness in humans (Hcfpl-CoVs) [9]. Both complete genomes of FECV and FIPV strains obtained in this study were detected as recombinants. Intra-species recombination events (fragments were all from FCoV strains) contribute to the generation of the FECV-25HZ-F652-Fec strain, while inter-species cross-over events (fragments were from FCoV and CCoV) play a role in the generation of the FIPV-25HZ-F653-Ple strain. These results provide direct evidence supporting the occurrence of cross-species transmission between FCoV and CCoV in Zhejiang province of China.

## 5. Conclusions

In this study, we developed an alternative RT-qPCR assay for the universal detection of FCoV and CCoV in 700 clinical samples collected from the Zhejiang–Jiangsu region of China in 2025. Both FCoV and CCoV were detected in different sample types, with ascitic and pleural fluids showing the highest positive rates. S gene and complete genome sequencing identified cross-species transmission and inter-species recombination events between the FCoV and CCoV strains obtained in this study. Overall, this study not only provides up-to-date epidemiological information on FCoV and CCoV in the Zhejiang–Jiangsu provinces of China but also confirms cross-species transmission and recombination between FCoV and CCoV strains in China.

## Figures and Tables

**Table 1:** Primers and probe for FCoV-CCoV RT-qPCR assay development and S gene amplification.

Primers and Probes	Sequences (5′ to 3′)	Position *	Reference
CoV-P0	FAM-AGGTACAAGCAACCCTA-MGB	29,117–29,113 bp	This study
CoV-F2	CTACTCTTGTACAGAATGGTAAGCACGT	29,082–29,108 bp	This study
CoV-R2	CTAAATCTAGCATTGCCAAATCAAATC	29,153–29,179 bp	This study
P205	GGCAACCCGATGTTTAAAACTGG	29,026–29,084 bp	Previous study [20]
P211	CACTAGATCCAGACGTTAGCTC	29,227–29,248 bp	Previous study [20]
CoV-S-F3	CAAAGATTTGAGTATTGGACTGTG	20,048–20,071 bp	This study
CoV-S-R2	GTATCACCAAAACCTATACACACAAG	24,924–24,949 bp	This study

* The position was determined according to the FIPV-25HZ-F653-Ple strain (GenBank no.: PZ518637).

**Table 2:** Repeatability and reproducibility of the universal FCoV-CCoV RT-qPCR assay.

Sample *	Intra-Repeatability	Coefficient of Variation (CV)	Inter-Reproducibility	Coefficient of Variation (CV)
10^7^ copies/μL	17.47 ± 0.04 ^#^	0.25%	17.54 ± 0.10	0.60%
10^5^ copies/μL	23.57 ± 0.09	0.39%	23.28 ± 0.24	1.07%
10^3^ copies/μL	30.69 ± 0.21	0.69%	30.84 ± 0.16	0.53%

* Different concentrations (10^3^~10^7^ copies/μL) of the standard plasmid were used. ^#^ Each mean Ct ± standard deviation was from three replicates.

**Table 3:** FCoV-CCoV detection in 700 clinical samples collected in 2025.

Year/Location/Sample Type	Feline No.	Feline Positive No.	Canine No.	Canine Positive No.	Total No.	Total Positive No.
**Location**						
Zhejiang	457	63 (13.78%)	198	12 (6.06%)	655	75 (11.45%)
Jiangsu	44	16 (36.36%)	1	1 (100%)	45	17 (37.77%)
**Sample variety**						
Serum	315	23 (7.30%)	148	9 (6.08%)	463	32 (6.91%)
Feces	113	33 (29.20%)	39	4 (10.25%)	152	37 (23.34%)
Nasal–oral swab	46	2 (4.34%)	12	0 (0%)	58	2 (3.44%)
Ascitic fluid	17	15 (88.23%)	0	0 (0%)	17	15 (88.23%)
Pleural fluid	10	6 (60%)	0	0 (0%)	10	6 (60%)
**Total**	501	79 (15.76%)	199	13 (6.53%)	700	92 (13.14%)

**Table 4:** Detailed comparisons of FIPV-25HZ-F653-Ple and FECV-25HZ-F652-Fec strains with representative FCoV strains.

Gene/Genome		ORF1ab		S	ORF3			E	M	N	ORF7		Genome
		1a	1ab		3a	3b	3c				7a	7b	
**Identity (%) to FIPV-25HZ-F653-Ple**	**BLACK**	93.7 ^a^	95.56	90.12	91.55	74.32	93.68	97.56	96.21	92.04	97.03	88.35	90.77
	**C1Je**	90.02	93.13	88.63	91.55	83.78	64.43	93.9	93.94	92.57	96.04	85.44	87.91
	**HF1902**	94.39	95.75	78.02	91.55	74.32	93.68	97.56	96.21	92.04	97.03	88.83	89.52
	**UG-FH8**	94.84	96.18	91.96	91.55	97.3	98.81	97.56	95.45	93.9	96.04	87.38	91.94
	**MW030110 SD**	94.49	95.97	91.76	90.14	93.24	98.42	96.34	94.7	93.63	99.01	90.29	91.37
	**JP15**	93.73	92.01	91.96	95.77	94.59	96.84	95.12	95.45	91.25	97.03	89.32	91.19
	**FIPV79-1146**	92.93	94.31	43.95	64.79	52.7	23.72	95.12	96.21	92.57	96.04	87.38	83.78
**Identity (%) to FECV-25HZ-F652-Fec**	**BLACK**	90.86	94.02	90.31	91.55	74.32	93.68	95.12	93.94	92.84	98.02	86.89	90.23
	**C1Je**	88.23	92.2	88.15	91.55	83.78	64.43	90.24	93.18	93.63	97.03	86.41	87.83
	**HF1902**	91.21	94.13	77.81	91.55	74.32	93.68	95.12	93.94	92.84	98.02	87.38	88.85
	**UG-FH8**	91.73	94.45	91.1	91.55	97.3	98.81	95.12	92.42	93.37	97.03	87.38	91.13
	**MW030110 SD**	91.31	94.29	92.02	90.14	93.24	98.42	91.46	92.42	94.16	100	88.83	90.86
	**JP15**	91.33	94.29	91.75	95.77	94.59	96.84	92.68	93.94	89.66	98.02	89.32	90.72
	**FIPV79-1146**	90.04	92.34	43.65	64.79	52.7	23.72	92.68	93.94	93.1	97.03	87.86	82.82
	**FIPV-25HZ-F653-Ple**	92.53	94.83	91.63	100	100	100	95.12	95.83	93.9	99.01	90.29	91.59

^a^ The numbers indicate the percentage (%) of similarity between each comparison.

**Table 5:** Summary of potential recombination events identified by RDP v.4.71.

Recombinant Virus	Parental Virus		Breakpoint *			Score for the Seven Detection Methods Embedded in RDP4						
	Major	Minor	Region	Begin	End	RDP	GENECONV	BootScan	MaxChi	Chimaera	SiScan	3Seq
FECV-25HZ-F652-Fec	QS2021	NLD/UU88/2010	ORF 1a	5970	8788	1.807 × 10^−12^	4.478 × 10^−6^	3.593 × 10^−12^	3.297 × 10^−2^	8.950 × 10^−4^	8.851 × 10^−14^	9.597 × 10^−3^
	HLJ/DQ/2016/01	HF1902	ORF 1b	13,914	15,042	1.643 × 10^−8^	1.294 × 10^−4^	2.223 × 10^−9^	2.024 × 10^−2^	- #	1.309 × 10^−7^	-
	QS2021	FIPV-25HZ-F653-Ple	ORF 3c E, M, N	25,412	27,350	2.542 × 10^−16^	2.966 × 10^−28^	-	6.225 × 10^−9^	5.433 × 10^−6^	7.824 × 10^−18^	-
FIPV-25HZ-F653-Ple	CU27528	ZJU1709	ORF 1a	1543	2230	9.262 × 10^−4^	-	9.901 × 10^−3^	2.100 × 10^−2^	6.078 × 10^−3^	5.090 × 10^−5^	-
	SD2021	ZJU1709	ORF 1a	2813	3696	2.903 × 10^−8^	9.993 × 10^−5^	-	4.077 × 10^−3^	1.154 × 10^−2^	6.292 × 10^−8^	-
	ZJU1709	UG-FH8	ORF 1a	4504	5090	8.766 × 10^−5^	-	-	1.619 × 10^−2^	-	1.700 × 10^−5^	-
	SD2021	NLD/UU88/2010	ORF 1a	7028	8220	3.381 × 10^−9^	-	3.615 × 10^−6^	8.768 × 10^−3^	4.281 × 10^−3^	6.254 × 10^−7^	1.029 × 10^−3^
	UG-FH8	CCoV 23/03	ORF 1b	14,006	15,644	1.086 × 10^−43^	1.111 × 10^−12^	8.133 × 10^−43^	3.383 × 10^−14^	1.658 × 10^−16^	1.491 × 10^−12^	1.022 × 10^−11^

* The breakpoint position in the genomes of the tested virus. # “-” indicates that the recombination event is not significant.

## Data Availability

The data presented in this study are openly available in GenBank, reference number PZ518627~PZ518638.

## Supplementary Materials

The following supporting information can be downloaded at https://www.mdpi.com/article/10.3390/vetsci13070661/s1: Table S1: Comparative detection of FCoV-positive samples using our assay and a previous method.
