# Supplementary material for: Cross-Species Transmission and Recombination Between Feline and Canine Coronaviruses in Jiangsu–Zhejiang Region in 2025
Source: Vet Sci. 2026 Jul 7;13(7):661. doi: 10.3390/vetsci13070661 (PMC13431536; doi:10.3390/vetsci13070661)
Supplement: Supplementary file 1 [file vetsci-13-00661-s001.zip › vetsci-4413333-supplementary.pdf]

Table S1 Comparative detection of FCoV-positive samples using our assay and a previous method.

Year/Location/sample type	Feline Sample no.	Our method Positive Sample no.	Previous method * positive Sample no.
**Location**			
Zhejiang	452	63 (13.9%)	44 (9.73%)
Jiangsu	49	16 (32.65%)	14 (28.57%)
**Sample**			
Serum	315	23 (7.3%)	9 (2.85%)
Feces	113	33 (29.2%)	31 (27.43%)
Naso-oral swabs	46	2 (4.34)	1 (2.17%)
Ascitic fluid	17	15 (88.23%)	11 (6.47%)
Pleural fluid	10	6 (60%)	6 (60%)
**Total**	501	79 (15.76%)	58 (11.57%)

* The previous method was from the reference (Herrewegh et al., 1995).
